# Effectiveness of Bortezomib in Cardiac AL Amyloidosis: A Report of Two Cases

**DOI:** 10.1155/2014/627474

**Published:** 2014-03-10

**Authors:** Santi Nigrelli, Giuseppe Curciarello, Piercarlo Ballo, Stefano Michelassi, Francesco Pizzarelli

**Affiliations:** ^1^Department of Medicine, Nephrology Unit, St. M. Annunziata Hospital, ASL 10, Florence, Italy; ^2^Hematology and Transfusional Service, St. M. Annunziata Hospital, ASL 10, Florence, Italy; ^3^Cardiology Unit, St. M. Annunziata Hospital, ASL 10, Florence, Italy

## Abstract

Cardiac involvement is a major prognostic determinant in patients with primary AL amyloidosis. The clinical results of standard therapeutic approaches are suboptimal. It has been recently shown that bortezomib, an inhibitor of the proteasome, can induce rapid favourable responses in AL amyloidosis improving cardiac function and survival. Herein we report on two patients with cardiac amyloidosis treated by bortezomib who experienced partial or total remission of hematologic disease and of cardiac involvement. However, death of one patient, suffering from chronic kidney disease stage 5, due to fulminant respiratory syndrome suggests the need for caution in bortezomib use if patients have this comorbid condition.

## 1. Introduction

Cardiac amyloidosis, which occurs as a complication of primary amyloidosis, is a result of cardiac deposition of insoluble, monoclonal immunoglobulin light chain fragments. This is observed in about 50% of patients with light chain amyloidosis predominantly after 40 years of age with higher prevalence in men than in women. The development of restrictive cardiomyopathy, complicated by progressive left ventricular or biventricular dysfunction, represents a major determinant of adverse outcome for these patients who typically die as a result of progressive heart failure or sudden cardiac death.

A simple staging index for cardiomyopathy, based on levels of N-terminal brain natriuretic peptide (NTproBNP) and troponin I at presentation of disease, has been shown to predict median survival of 27.2, 11.1, and 4.1 months in stages I (normal values of both markers), II (altered values of only one marker), or III (altered values of both markers), respectively [1].

The aim of cardiac amyloidosis treatment is to reduce the concentration of the amyloidogenic light chains and to induce improvement of cardiac function and survival [2, 3] and it is crucial that treatment is rapidly effective, tolerable, and safe.

The severity of cardiac involvement and the high toxicity treatment profile, however, have always limited the outcome of traditional treatment options based on high dose melphalan chemotherapy followed by autologous stem cell transplantation.

Recently it has been shown that bortezomib, a proteasome inhibitor, can induce rapid responses in AL amyloidosis improving cardiac function and survival [4].

We report herein two cases of cardiac amyloidosis in which treatment with bortezomib was associated with partial or complete clinical remission of disease.

## 2. Case Presentation

### 2.1. Case Report 1

A 54-year-old man was referred to our institution in June 2006 because of clinical signs of congestive heart failure.

He was a long distance runner and diver but during the last year he had experienced progressive dyspnea and fatigue in his sport activities.

Clinical examination at admission showed breathlessness, peripheral edema, distended neck veins, hepatic engorgement, and upper quadrants abdominal pain. Clinostatic arterial pressure was 110/70 mmHg with a heart rate of 80 bpm, and orthostatic arterial pressure was 100/70 mmHg with heart rate of 84 bpm.

**Figure 1:**
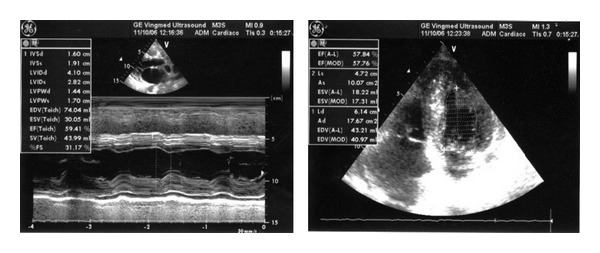
Case 1: echocardiography before bortezomib.

The electrocardiogram showed diffuse low-voltage QRS complexes. Echocardiography showed a left ventricle with normal diameters (end diastolic diameter 41 mm, end systolic diameter 28 mm) and thickened walls (Table 1) and preserved systolic function (EF 59%) but typical “sparkling” texture of the myocardium and a restrictive transmitral pattern (E/A = 3.9) suggestive of advanced left ventricular diastolic dysfunction. Atrial enlargement (43 mm) and mild pericardial effusion were also found (Figure 1). High serum and urinary monoclonal  *κ*  light chain was found with high-serum-free  *κ*/*λ*  ratio, as were high levels of serum NTproBNP (5570 ng/mL) and troponin I (0.85 ng/mL) (Table 1). The glomerular filtration rate was normal and the daily proteinuria was absent. Periumbilical fat aspirate sample confirmed amyloidosis.

A bone marrow biopsy showed 25% of plasma cells with interstitial/paratrabecular distribution. K plasma cellular clonal restriction was observed.

Abdominal ultrasound, EMG, nerve conductivity, and total body bone radiographs were normal. A genetic study excluded a transthyretin amyloidosis.

From August 2006 to March 2007, the patient received a total of 6 cycles of melphalan (14 mg/day) and dexamethasone (20 mg/day) for 4 days. The therapy was frequently discontinued because of recurrent leucopenia.

At the end of this period, blood examination showed a significant decrease in free serum *κ* light chains but NTproBNP and troponin I levels were increased (Table 1) and the patient remained ill.

From April to June 2007 the patient performed 3 cycles of therapy with bortezomib (1.3 mg/sqm/day) and dexamethasone (20 mg/day) administered on the 1st, 4th, 8th, and 11th day of every 21 days course followed by 8 infusions of bortezomib (1.3 mg/sqm/day) every 10 days and 3 infusions every 20 days. The treatment was well tolerated and was stopped in January 2008.

Since the first cycle of bortezomib, the patient experienced a progressive improvement in breathlessness with complete remission during the treatment.

**Figure 2:**
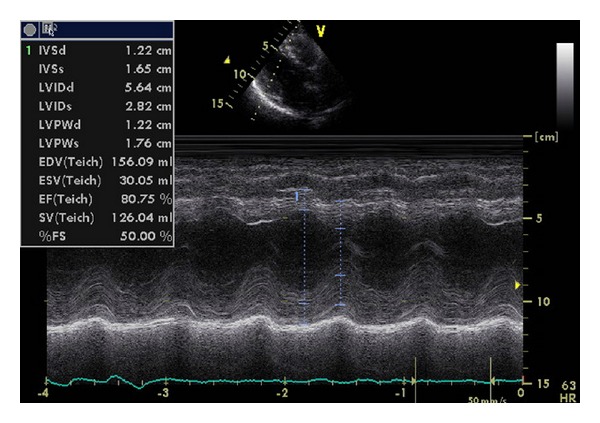
Case 1; echocardiography after bortezomib.

At the end of therapy serum free *κ* light chains and free  *κ*/*λ*  ratio were normal while NtproBNP and troponin I were markedly reduced. Moreover, a considerable reduction in left ventricular wall thicknesses was found (Table 1, Figure 2). During the following years, all echocardiographic indexes progressively returned to normal values and complete remission of haematological disease was achieved.

In September 2013, the patient was still asymptomatic and routinely performed his usual sport activities without any significant physical limitations. Serum *κ* chains (11 mg/dL) NTproBNP (204 ng/mL) and troponin I (0.05 ng/mL) concentrations were normal. Echocardiographic indexes were all within normal ranges, and in periumbilical fat aspirate sample amyloid deposits disappeared (Table 1).

Based on these descriptors, we can consider the patient recovered.

### 2.2. Case Report 2

A 67-year-old golf instructor was admitted to our unit in January 2005 because of recent onset of nephrotic syndrome.

His medical history highlighted renal stones, relapsing bladder papillomatosis, and a progressive, unintentional weight loss (about 20 kg) that occurred between 2003 and 2004.

On clinical examination diffuse edema and liver and spleen enlargement were observed. Arterial pressure and heart rate were normal.

Blood tests on admission showed serum creatinine levels of 1.33 mg/dL, total serum protein 5.6 gr/dL, monoclonal gammopathy IgGk, YGT 225 U/L (<66 U/L), alkaline phosphatase 253 U/L (<129 U/L), and NT proBNP 1102 pg/mL (n.v. < 227 pg/mL). Urine tests showed daily proteinuria of 16 gr and Bence Jones proteinuria with monoclonal *κ* light chains (Table 2).

Periumbilical fat aspirate sample showed amyloidosis confirmed by kidney biopsy. Amyloid deposits were identified by optical microscopy in glomerular mesangium and renal arteriolar walls. Further immunofluorescence microscopy showed monoclonal *κ* chain deposits in mesangium and capillary walls.

A bone marrow biopsy showed an interstitial plasmacytosis (10%) with clonal plasma cells restriction for *κ* chains.

Echocardiography showed mild hyperechogenicity of left ventricular myocardium without thickening of walls.

A diagnosis of AL amyloidosis with involvement of kidney, liver, and heart was made.

In February 2005, the patient started on treatment with melphalan (16 mg for 4 days) and prednisone (75 mg for 7 days) every 6 weeks. Only two cycles of chemotherapy were performed because in April 2005 the patient experienced acute renal failure (serum creatinine up to 12 mg/dL with only partial resolution to serum creatinine 5.7 mg/dL at discharge) and severe arterial hypertension. Only mild reduction of serum free *κ* light chains was observed with no remission of nephrotic syndrome (Table 2).

From July to December 2005, the patient received oral dexamethasone (40 mg for 4 days/monthly) and from September 2005 the patient received also thalidomide (200 mg/day). Treatment continued but was interrupted between January and May 2006 due to serious polyneuropathy, relapsing pulmonary infection, and herpes zoster infection.

In May 2006, the patient progressively developed clinical signs of heart failure and terminal uremia requiring hemodialysis: thalidomide (200 mg/day) was restarted at this point.

**Figure 3:**
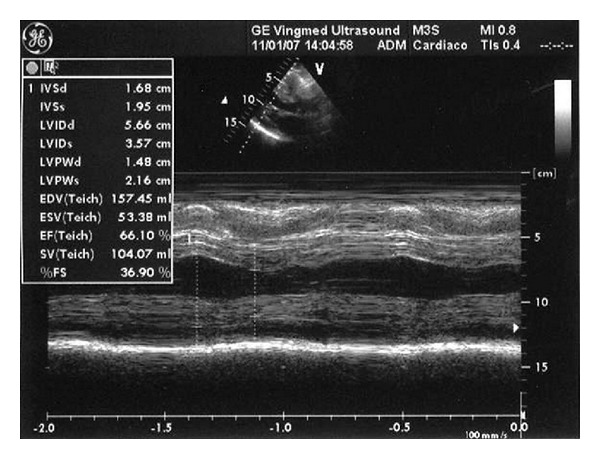
Case 2; echocardiography after the melphalan/dexamethasone cycles.

After four months, echocardiography showed increased wall thickness (Figure 3), and the patient experienced worsening of heart failure (NYHA class III) and an increase in both free serum *κ* light chains and BNP. Following this, the patient received 3 cycles of treatment with melphalan (12 mg/day) and dexamethasone (20 mg/day) for 4 days every 28 days without clinical results (Table 2).

Clinical conditions, echocardiographic indexes, and laboratory examination worsened further during the following months. A 35-day cycle with bortezomib (1.3 mg/sqm) i.v. and dexamethasone (20 mg/day) on the 1st, 8th, 15th, and 22nd days was then started.

**Figure 4:**
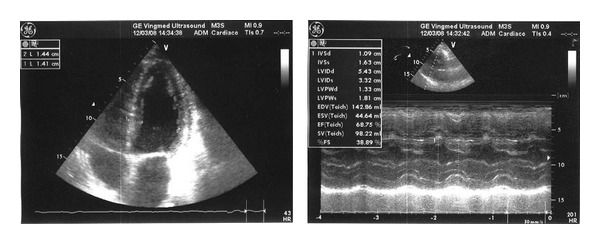
Case 2: echocardiography after the bortezomib cycles.

After 6 cycles, June 2008, the patient experienced improvement of general conditions and returned to work. Serum free *κ* ligth chains, *κ*/*λ* ratio and BNP levels were markedly reduced (Table 2), whilst echocardiography showed a significant reduction in left ventricular wall thickness (Figure 4). Treatment was interrupted.

**Figure 5:**
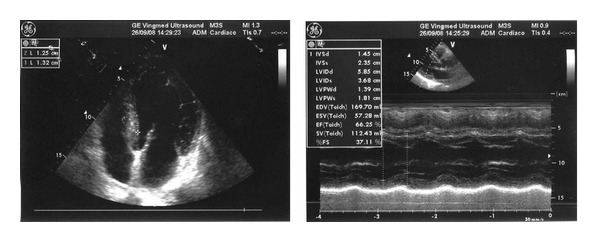
Case 2: echocardiography before the last bortezomib cycle.

In October 2008, because of progression of organ damage (Figure 5) and increase of serum free *κ* light chains (Table 2), the patient restarted therapy with bortezomib and dexamethasone. Unfortunately, 10 days after the end of the first cycle, the patient died because of a fulminant syndrome characterized by diffuse infiltrative pulmonary disease with severe respiratory failure, severe hypotension, and lactic acidosis (up to 10.8 mmol/L) without signs of infections.

## 3. Discussion

Until some years ago, treatment of AL amyloidosis consisted only of oral administration of melphalan/prednisone in repeated cycles over several months. The treatment rarely fully eliminated production of monoclonal light chains, and its impact on survival was poor.

A more aggressive approach consisting of melphalan administered in myeloablative doses followed by autologous stem cell transplantation (HDM/SCT) began to be used in the mid-19 90s. Early experiences were encouraging but autologous stem cell transplantation (ASCT) related mortality (21% even at referral centers) and a high toxicity profile limited the practical feasibility of this approach to selected patients.

An alternative treatment consists of oral melphalan associated with dexamethasone in 4-day cycles each month (MDex) [5, 6]. Early reports of this treatment describe rapid eradication of monoclonal light chain production and improvement in NTproBNP. Unfortunately the need for repeated courses with high dose dexamethasone increases the risk of fluid retention, cardiac failure, and myopathy. Moreover, duration of hematologic response with this treatment is unclear.

Recently identified alternatives to melphalan based treatment such as thalidomide or lenalidomide (a thalidomide analog) combined with dexamethasone may lead to hematologic response in some individuals with persistent plasma cell dyscrasia after initial treatment with HDM/SCT or Mel/Dex; however, the high dose needed to obtain hematologic response and frequent peripheral persistent neurotoxicity discourage their extensive use [7].

In small retrospective studies, bortezomib, a boronic acid dipeptide, reversible inhibitor of the 26S proteasome, was proven to be able to induce a high rate of rapid response in patients with AL amyloidosis [8]. The high activity of bortezomib in AL amyloidosis could be due to an imbalance between proteasome load and capacity favored by the peculiar conformational instability of amyloidogenic light chains [9, 10].

A large retrospective study of 94 patients with AL amyloidosis treated with bortezomib and dexamethasone confirmed the efficacy of this combination and showed a significant improvement of cardiac performance among patients with heart involvement [4]. The safety and efficacy of bortezomib as a single agent in AL amyloidosis have been prospectively evaluated in a multicenter trial [11].

The clinical course of our two patients after bortezomib treatment deserves remarks.

- Both patients received previous treatment with melphalan, dexamethasone, and thalidomide and experienced some significant reduction, if any, of serum light chains but without improvement of cardiac failure.
- Both patients just after the first cycle of bortezomib experienced a rapid significant reduction of serum free light chains and of NTproBNP or BNP associated with improvement of signs of cardiac failure and followed by reduction of cardiac walls thicknesses.The depressive effect of high concentration of light chains on heart function is common knowledge but in our patients speed of reduction appears more important for improvement of cardiac function than absolute light chains concentration reduction (obtained also with traditional therapy but more slowly).
- In our patients, cardiac recovery of normal morphology was obtained later after the end of bortezomib therapy supporting the hypothesis of an indirect effect of bortezomib on tissutal environment whereby solubility or disappearance of amyloid systemic tissutal deposits is favored as shown by normal myocardium wall thicknesses and by lack of amyloid deposits in periumbilical fat 6 years later. This observation is indeed uncommon: there does not exist a similar case even in the large retrospective study of Kastritis et al. [4].
- Though bortezomib/dexamethasone combination seems to fulfill many requirements for optimal treatment of AL amyloidosis, polycentric observational study evidenced a high rate of discontinuation of bortezomib (39% of study population) due to adverse events, sometimes as severe as death [9]. Diffuse infiltrative lung disease represents a fatal, uncommon, unexplained adverse event that correlates to use of the drug probably favoured by comorbid conditions. Patient 2 had no previous history of pulmonary involvement but was on hemodialysis and showed an important liver involvement. We speculate that these two conditions are predisposing risk factors for diffuse infiltrative pulmonary disease. Other studies are needed to better define the safety profile of bortezomib in comorbid subjects especially when renal or liver functions are compromised.

## Figures and Tables

**Table 1:** Followup of case report 1.

	June 2006 Basal	March 2007 after mel/dex	January 2008 after bort/dex	April 2008	October 2008	April 2009	September 2009	September 2013
Serum free *k* light chains mg/dL (n.v. < 19.4 )	292	110	10.6	12.07	12.03	12.09	13.05	7

*k*/*λ* ratio (0.26–1.65)	56.1	32.16	1.1	1.01	1.00	2.01	0.94	

NTproBNP ng/mL (n.v. < 227)	5570	8593	840	695	521	482	423	204

Troponin I ng/mL (n.v. < 0.06)	0.85	3.23	0.59	0.83	0.85	0.19	0.19	0.05

IVS (mm)	16	16	14	14	12	12	12	11

LVPW (mm)	16	15	14	14	12	12	12	11

mel: melphalan; dex: dexamethasone; bort: bortezomib.

**Table 2:** Followup of case report 2.

	January 2005 Basal	April 2005 after melp/pr 2 cycles	September 2006 after dex/thalidomide	January 2007 after 2 cycles of melp/dex (Figure 3)	July 2007 before bort/dex	June 2008 after bort/dexa 6 cycles (Figure 4)	October 2008 (Figure 5)
Serum free *k* light chains mg/dL (n.v. < 19.4 )	92	63	174	134	331	149	189

*k*/*λ* ratio (0.26–1.65)	6.13	2.92	2.95	4.4	7.4	4.53	5.57

BNP pg/mL (n.v. < 50)		81	2976	2480	3138	484	1292

Troponin I ng/mL (n.v. < 0.06)		0.05	0.04	0.02	0.04	0.04	0.07

IVS (mm)	9	11	13	17		12	14

LVPPW (mm)	9	10	13	15		12	13

mel: melphalan; pr: prednisone; dex: dexamethasone; bort: bortezomib.
